# TCR-like antibodies targeting autoantigen-mhc complexes: a mini-review

**DOI:** 10.3389/fimmu.2022.968432

**Published:** 2022-07-27

**Authors:** Ying Li, Wei Jiang, Elizabeth D. Mellins

**Affiliations:** ^1^Department of Pediatrics, Divisions of Human Gene Therapy and Allergy, Immunology & Rheumatology, Stanford University School of Medicine, Stanford, CA, United States; ^2^Stanford Program in Immunology, Stanford University School of Medicine, Stanford, CA, United States

**Keywords:** TCR-like antibodies, autoimmune diseases, autoantigen presentation, immunotherapy, antigen-specific therapy

## Abstract

T cell receptors (TCRs) recognize peptide antigens bound to major histocompatibility complex (MHC) molecules (p/MHC) that are expressed on cell surfaces; while B cell-derived antibodies (Abs) recognize soluble or cell surface native antigens of various types (proteins, carbohydrates, etc.). Immune surveillance by T and B cells thus inspects almost all formats of antigens to mount adaptive immune responses against cancer cells, infectious organisms and other foreign insults, while maintaining tolerance to self-tissues. With contributions from environmental triggers, the development of autoimmune disease is thought to be due to the expression of MHC risk alleles by antigen-presenting cells (APCs) presenting self-antigen (autoantigen), breaking through self-tolerance and activating autoreactive T cells, which orchestrate downstream pathologic events. Investigating and treating autoimmune diseases have been challenging, both because of the intrinsic complexity of these diseases and the need for tools targeting T cell epitopes (autoantigen-MHC). Naturally occurring TCRs with relatively low (micromolar) affinities to p/MHC are suboptimal for autoantigen-MHC targeting, whereas the use of engineered TCRs and their derivatives (e.g., TCR multimers and TCR-engineered T cells) are limited by unpredictable cross-reactivity. As Abs generally have nanomolar affinity, recent advances in engineering TCR-like (TCRL) Abs promise advantages over their TCR counterparts for autoantigen-MHC targeting. Here, we compare the p/MHC binding by TCRs and TCRL Abs, review the strategies for generation of TCRL Abs, highlight their application for identification of autoantigen-presenting APCs, and discuss future directions and limitations of TCRL Abs as immunotherapy for autoimmune diseases.

## Introduction

To date, over 80 autoimmune diseases have been described (1), ranging from organ-specific (e.g., pancreas-specific Type 1 diabetes (T1D) and thyroid gland-specific Grave’s disease) to systemic conditions (e.g., rheumatoid arthritis (RA) and systemic lupus erythematosus (SLE)). Curative approaches for autoimmunity are lacking. Despite diverse manifestations and autoantigen sources, these autoimmune reactions typically share stages of initiation, propagation, and for some, periods of clinical remission (2).

Although environmental factors are thought to be required as triggers for disease, predisposition to autoimmunity most often reflects inherited factors, with MHC (human leucocyte antigen (HLA) in humans) alleles conferring the highest risk (3, 4). Typically, class I (MHC-I or HLA-I in humans) genes encode proteins that present peptides from intracellular antigens to CD8+ T cells, and class II (MHC-II or HLA-II in humans) genes encode proteins that present extracellular/endosomal antigens to CD4+ T lymphocytes (5). A number of particular HLA-II (i.e., HLA-DR, -DQ, and -DP) alleles have been identified as critical risk factors for particular autoimmune diseases. For example, >90% celiac patients carry HLA-DQA1*05:01/HLA-DQB1*02:01 (6, 7), and >95% narcoleptic patients carry HLA-DQB1*06:02 (8, 9). In addition, the HLA-DRB1*04:01/*04:04 genotypes are risk alleles (odds ratios are ∼4.14 and ∼3.17, respectively) for RA (10) and the HLA-DRB1*15:01-DRB5*01:01 haplotype (up to 60% among Caucasians) is linked to multiple sclerosis (MS) (11). How these polymorphic MHC proteins interact with autoantigens and how autoantigen-MHC presenting APCs interact with autoreactive T cells are central questions in the field.

HLA-II+ APCs generate peptide/HLA-II (p/HLA-II) complexes (12, 13) that interact with cognate TCRs on CD4+ T cells, which orchestrate downstream autoimmune reactions (9, 14–16). Therefore, targeting autoantigen-HLA-II complexes on the APC surface with soluble TCR or TCRL reagents enables a specific way to investigate the initiation and propagation of autoimmunity. Here, we review current approaches and future directions for generating and using TCRL (also known as TCR mimic) Abs as research tools and potential therapeutics for autoimmune diseases.

## Comparisons of TCRL Abs with TCRs

Abs share many similarities with TCRs in terms of diversity of the receptor repertoire and specificity for antigen recognition (17, 18). Abs, especially monoclonal Abs (mAbs) are widely used in research, diagnoses and therapies as specific immune-targeting agents (19), whereas TCRs have not been widely used (20). This is in large part due to the intrinsic difference in their antigen binding affinities. TCRs have micromolar affinities for cognate p/MHCs (21), whereas Abs have nanomolar affinities and interact with their specific antigens with >100x higher binding energies (22).

Each TCR contains two polypeptide chains: α and β, whereas each Ab consists of two heavy (H) and two light (L) chains. An Ab has two identical antigen-binding fragments (Fab, an H/L dimer) and a crystallizable fragment (Fc, from the H chain) that links the two Fab arms (22), yielding increased avidity for antigen. The Fab H/L heterodimer, like the TCR α/β heterodimer, uses two sets of complementarity-determining regions (CDRs) to directly contact the cognate antigen. The CDR regions are also referred to as the fragment variable (Fv) region. CDR3 of both Fabs and TCRs are hypervariable, with key amino acid residues governing antigen binding specificity. Residues within the germline-encoded CDR1 and CDR2 are less variable (17). Ab engineering usually focuses on CDRs of Fab or Fv heavy and/or light chains. To modify both chains using one gene cassette, a covalent link between the heavy and light chain fragments can be used, yielding single-chain Fv (scFv) for example.

As natural p/MHC receptors, TCRs have scientific, diagnostic and therapeutic potential, particularly if used as tetramers or higher order multimers to increase avidity (23), or if engineered to improve target affinity or avidity (23–25). Affinity improved and/or multimeric TCRs and TCR-engineered T cells have been used to target and clear tumor cells presenting cancer-related p/MHC-I (26). However, these reagents have seldom been used for autoantigen-MHC-II targeting, likely for several reasons. First, compared to TCRs recognizing foreign or neoantigens, MHC-II/autoantigen-reactive TCRs tend to have lower affinity, which typically allows their escape from thymic negative selection but activity for autoimmune responses (27); this affinity window is a poor starting point for affinity improvement by TCR engineering. Second, improved TCR affinity is often compromised by unpredictable cross-reactivity (28, 29), causing off-target staining during auto-APC characterization.

To resolve these issues stemming from natural TCRs, investigators developed TCRL mAbs by combining the high affinity of a mAb with the capacity to recognize p/MHC complexes (20). Some TCRL mAbs target intracellular antigens presented by MHC-I on tumor cells and have been applied as immunotherapeutics for cancers (30, 31). Crystallization studies have determined the structures of five p/MHC-I-specific TCRL mAbs in Fab formats binding to their p/MHC-I targets (32–35). Although CDR regions of all five TCRL Fab molecules interact with the peptide region of p/MHC-I complexes, only two (34) show the canonical docking geometry of TCRs with p/MHC (20). Thus, the TCR docking geometry that elicits TCR signaling (36) is not an absolute requirement for TCRL mAb development. Recently, the co-crystal structure of an MHC-II-restricted TCRL Fab bound by a gliadin peptide/HLA-DQ2.5 (DQA1*05:01/DQB1*02:01) complex has been determined (37). This Fab has picomolar affinity, adopts the canonical TCR docking geometry (38), and demonstrates desirable properties for p/MHC-II staining and specific T cell inhibition relevant to celiac disease (37).

## Generation of TCRL mAbs targeting p/MHC-II complexes

Naturally occurring Abs rarely mimic TCR specificity for p/MHC antigen(s); therefore, the TCRL feature of an Ab is typically obtained through target-driven *in vitro* selection and/or Ab engineering. Advances in hybridoma technology (39), recombinant p/MHC synthesis (40), and binder selection *via* phage or yeast display (41, 42) have enabled protein engineering of TCRL mAb. As other reviews have summarized TCRL mAb generation (20, 30, 31), we focus on the available approaches relevant to TCRL mAbs specific for p/MHC-II.

Initially, mice or rats immunized with p/MHC-II complexes expressed by cells or as soluble, recombinant proteins were used to produce a candidate B cell pool from which B cell hybridomas (immortal B cell lines producing candidate mAbs) were generated. Although TCRL specificity was possible (43, 44), most often, p/MHC-II-specific enrichment and screening were required to identify hybridomas producing TCRL mAbs. To date, >20 p/MHC-II-specific TCRL mAbs have been generated using this approach (20, 45, 46) and about half are relevant to autoimmune diseases (**Table 1**). However, challenges persist: 1) limited B cell clonal candidates with peptide specificities and more clones with monomorphic MHC specificity due to the framework differences of MHC-II alleles or MHC-II from different species (immunization of HLA-transgenic mice (49) may help enrich for peptide-specific responses, see discussion below); 2) low throughput of hybridoma production and labor-intensive screening for p/MHC-II binding; 3) non-human origin of the Ab itself, limiting their therapeutic use. Notably, a human B cell hybridoma expressing a TCRL mAb recognizing an HLA-A2-derived self-peptide bound to HLA-DR1 was generated using peripheral blood mononuclear cells (PBMC) (59).

**Table 1:** TCRL mAbs targeting autoimmunity-related p/MHC-II complexes.

mAb Clone	Species	Format	Method	Disease/Model	T cell antigen/MHC	References
B-7-1, B-18-7, C-34-72	Mouse	Full-length Ab	Hybridoma	MS/EAE model	MBP_87-99_/I-A^s^	(47)
S.1.6	Mouse	Full-length Ab	Hybridoma	MS	MBP/DR7	(48)
R.1.D12	Mouse	Full-length Ab	Hybridoma	MS	MBP/DRw11	(48)
MK16	Mouse	Fab	Phage display	MS	MBP_218-231_/DR15	(49)
12A	Mouse	Full-length Ab	Hybridoma	RA	HC gp-39_263-275_/DR4	(50, 51)
2E4, 1F11, 2C3, 3A3, 3H5	Human	Fab	Phage display	MS	MOG_35-55_/DR15	(52)
G3H8	Human	Fab; reconstructed full-length Ab	Phage display	T1D	GAD65_555-567_/DR4	(52, 53)
mAb287	Mouse	Full-length Ab	Hybridoma	T1D/NOD mice	Insulin B_9-23_/I-A^g7^	(54, 55)
FS1	Mouse	Full-length Ab	Hybridoma	Diabetes/NOD mice	p63/I-A^g7^	(46)
106, 107	Human	scFv; reconstructed full-length Ab	Phage display	Celiac Disease	glia-α1a/DQ2.5	(56)
mAb757	Mouse	Full-length Ab	Hybridoma	T1D/NOD mice	Insulin B_9-23_/I-A^g7^	(57)
3-5	Mouse	Full-length Ab	Hybridoma	T1D/NOD mice	2.5HIP/I-A^g7^	(58)
206, 3.C11	Human	scFv; reconstructed full-length Ab	Phage Display	Celiac Disease	glia-α2/DQ2.5	(37)
**Selected other TCRL mAbs mentioned in this mini review**						
Y-Ae	Mouse	Full-length Ab	Hybridoma	Self-antigen	Eα/I-A^b^	(43, 44)
UL-5A1	Human	Full-length Ab	Hybridoma^*^	Self-antigen	HLA-A2_105-117_/DR1	(59)
I-5	Mouse	Full-length Ab	Hybridoma	Self-antigen	CLIP/DR3	(60)
D-4, G-32, and G-35	Mouse	Full-length Ab	Hybridoma	Model antigen	MCC/I-E^k^	(61, 62)
3M4E5 and 3M4F4	Human	Fab	Phage Display	Tumor antigen	NY-ESO-1/A*0201	(34)
13.4.1	Mouse	Fab	Phage Display	Viral antigen	HA_255-262_/H-2K^k^	(63)

^*^Human hybridoma. Note: See (20, 30, 31, 45, 46, 64–69) for a more comprehensive list of other TCRL mAbs, including anti-p/MHC-I reagents.

To avoid the limitations of hybridoma approaches, phage display has been applied by several groups to screen Ab libraries for p/HLA-II binders (49, 52, 56) (**Table 1**). A typical library contains 10^8^-10^11^ phage particles, each displaying an Ab variant on the surface. Phage display is achieved by covalently fusing Ab fragments, such as Fab and scFv, with a phage coat protein through molecular cloning (41, 70). Screening the library for binders to p/HLA-II relies on a process called “panning” or more recently “biopanning” (70). This process includes multiple rounds of negative selection (e.g., against irrelevant p/HLA-II) and positive selection (e.g., against target p/HLA-II). Designing Ab libraries in phage allows selection from mouse (49) or human (37, 52, 53, 56) antibody sources. To enrich for peptide-specific Abs in the mouse endogenous repertoire prior to construction of a phage-Fab library, the Fugger group immunized HLA-DR15 (DRA*01:01/DRB1*15:01) transgenic mice using DR15 molecules in complex with a myelin basic protein (MBP) peptide, leveraging the inherent DR15 tolerance of the model to skew the Ab response towards specificity for the MBP peptide (49). HLA-transgenic animal immunization followed by screening yielded a series of TCRL reagent findings, including the MBP/DR15-restricted TCRL mAb MK16 as mentioned (49), invariant chain peptide/HLA-DR mAb in another study (60), and an MHC-I-restricted TCRL mAb in additional work (63). Human Fab or scFv libraries built and expressed in phage have been mostly from large naïve repertoires (37, 52, 53, 56), which likely harbor TCRL candidates, albeit rare. Using stringent phage panning strategies, the Reiter and the Løset groups isolated DR-restricted (52, 53) and DQ-restricted (37, 56) human TCRL mAbs, respectively (**Table 1**). As these human Fabs or scFvs were not raised or matured against the target p/HLA-II, their affinities were suboptimal. Reconstructing a full-size Ab using the TCRL Fab or scFv increased the binding strength (37, 53). However, further affinity maturation may be useful. Recently, Frick et al. suggested a strategy to improve binder affinity *via* multiple rounds of phage-Ab library optimization and selection (37).

Combining phage display with yeast display is particularly useful for developing high affinity TCRL mAbs (71). Since first developed (42), yeast display technology has evolved, allowing surface display of monomeric or dimeric protein scaffolds (72, 73). Thus, either scFv or Fab identified from a phage-Ab library can be affinity matured using the yeast platform. Advantages of yeast display include 1) eukaryotic gene transcription and protein expression machinery for appropriate Ab folding; and 2) quantitative flow cytometry-based screening, ensuring high throughput selection for high-affinity Abs (74, 75).

## TCRL mAbs as research tools and therapeutics for autoimmune diseases

### Characterization of autoantigen-presenting APCs using TCRL mAbs

Presentation of autoantigen by APCs, especially professional MHC-II+ APCs, such as dendritic cells (DCs), macrophages (MФs), and B cells, is critical for CD4+ T cell activation and differentiation into helper T effector (T_eff_) or suppressive T regulatory (T_reg_) cells during autoimmune responses. An imbalance of T_eff_ and T_reg_ functions upon autoantigen recognition is believed to drive the loss of tolerance, with subsequent autoreactive T cell responses and production of autoantibodies (2). Therefore, the study of MHC-II+ autoantigen presenting cells (auto-APCs) is fundamental for understanding disease pathogenesis and may lead to novel immunotherapies. Murine models allow direct evaluation of tissue-resident and circulating APC subsets and enable genetic modifications of these APCs to assess their autoreactive functions. For example, using an experimental autoimmune uveitis (EAU) mouse model, Lipski et al. analyzed disease-related infiltrating MФs and resident retinal microglia by tissue immunostaining and cytometry-based immunophenotyping of isolated cells (76). In another model, single-cell sequencing was used to characterize tissue-infiltrating APCs in autoimmune diabetes (77). However, discoveries in murine models are not easily transferable to human diseases (78, 79).

Auto-APC identification using human samples is a preferred approach for clinical relevance. Early studies with human samples focused on APC enumeration in PBMC and biopsies. Increased frequency of circulating DCs was implicated in regulation of antigen presentation by islet cells and activation of autoreactive CD4+ T cells (80, 81). Recently, a novel approach was developed using PBMC to identify autoantigen-specific memory B cells, which are potent MHC-II+ APC (82). Therapeutic strategies focusing broadly on APC function have been developed, such as B cell depletion in SLE (83) and tolerogenic DC adoptive therapy (84). However, the key to the optimization of APC-directed immunotherapy is identification of autoantigen specificity.

Due to their high affinity and the ease with which they can be further engineered, TCRL mAbs have gradually replaced TCR-derived reagents in research and therapeutic development for autoimmunity. TCRL MK16, described above, identified microglia/MФs rather than astrocytes as the predominant auto-APCs in MS lesions (49). Human cartilage glycoprotein (HC gp-39, residues 263-275) represents a candidate T cell autoantigen in RA and can be presented by the RA susceptibility allele, HLA-DR4 (DRA*01:01/DRB1*04:01) (50, 85). TCRL mAb 12A specific for gp-39 (263-275)/DR4 identified autoantigen-presenting DCs in synovial tissue of DR4+ patients, indicating local presentation of gp-39 in inflamed joints (50, 51). Recently reported are several TCRL mAbs, specific for different gluten-derived peptide epitopes in complex with the celiac disease risk allele, HLA-DQ2.5. These complexes are known to be recognized by CD4+ T cells that drive disease (16, 38). The TCRL mAbs identified plasma cells, an unexpected APC, as the most abundant cell type presenting gluten peptides in gut biopsies from celiac patients (37, 56).

Although murine models cannot directly identify auto-APCs that function in human diseases, applying TCRL mAbs in these models may shed mechanistic light on disease pathology. For example, with specificity for a model antigen, moth cytochrome c-derived peptide (MCC, residues 95-103) bound by mouse MHC-II I-E^k^, TCRL mAb determined that a minimum of 200–400 p/MHC-II complexes per APC was necessary for T-cell stimulation (61). This number is at least an order of magnitude higher than the minimum requirement of p/MHC-I complexes for licensing cytolytic activity of human CD8+ T cells (86, 87).

### Therapeutic potential of TCRL mAbs in autoimmune diseases

**Figure 1:**
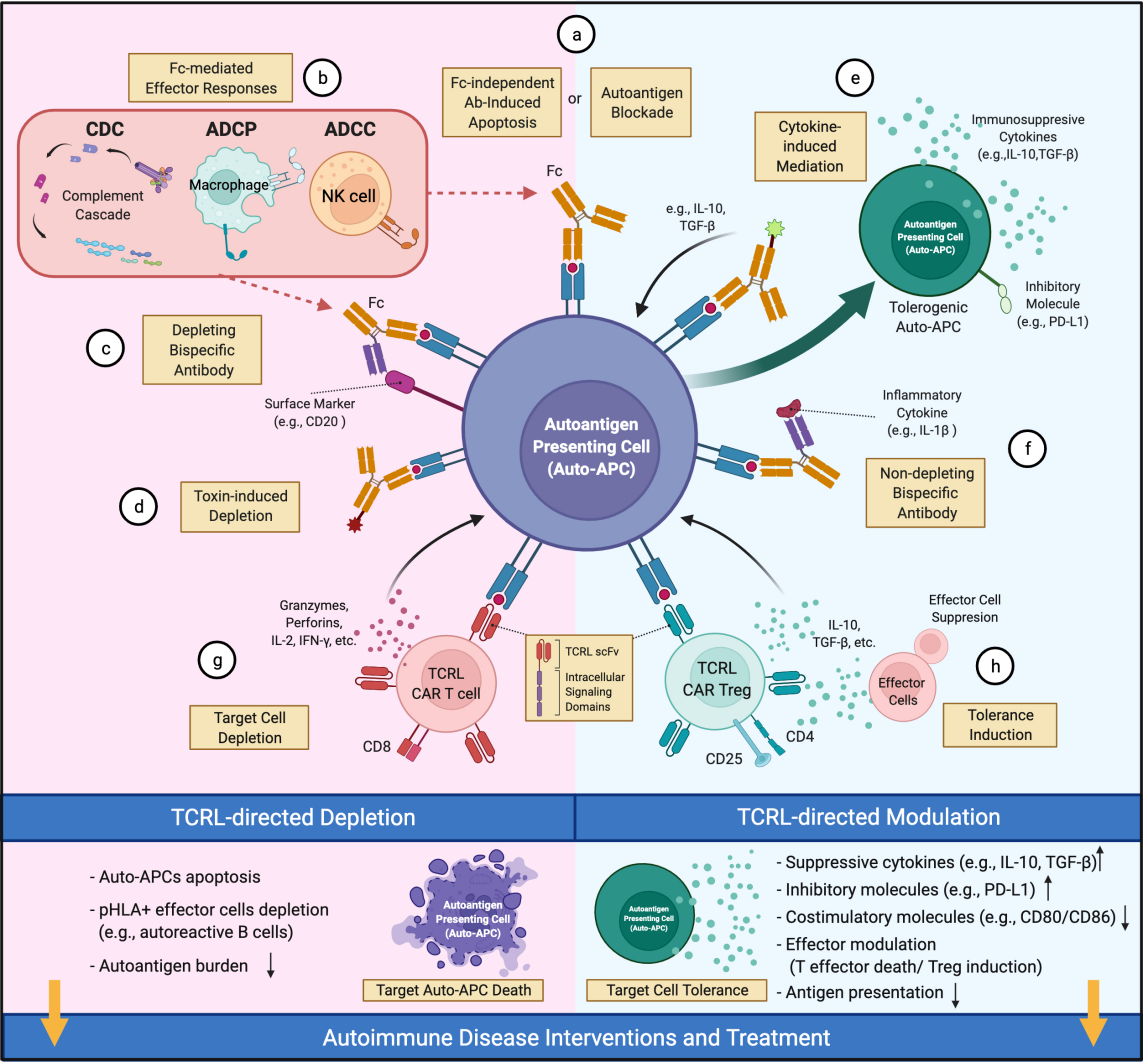
Therapeutic potential of TCRL mAbs in autoimmune diseases. TCRL mAbs specific for autoantigen/HLA complexes can elicit therapeutic effects *via* depleting (pink) or non-depleting (cyan) mechanisms. **(A)** TCRL mAbs either block autoantigen presentation or induce apoptosis of target cells. **(B)** TCRL mAbs induce Fc-mediated cytotoxicity through various effector mechanisms. **(C, F)** Bispecific antibodies targeting autoantigen/HLA complexes and either a surface marker of target cells or a pathogenic-related cytokine; **(D)** TCRL mAb–toxin conjugates induce auto-APC depletion by payload effector molecules, including cytokines, toxins or radioactive substances. **(E)** TCLR mAb-cytokine conjugates guide the delivery of immunomodulatory cytokines (e.g., IL-10, TGF-β) to auto-APCs for tolerance induction. **(G)** TCRL scFv fragments are reformatted into CARs for auto-APC targeting and depletion. **(H)** CD4+CD25+ TCRL CAR T_reg_ cells suppress T_eff_ function and induce tolerance. (This figure was created with BioRender.com).

TCRL mAbs have not been intensively investigated as therapeutics for autoimmune diseases, although their pre-clinical examination in cancers (30, 31) suggests therapeutic potential. In cancer, TCRL mAbs can target intracellular tumor antigens presented by cell surface MHC-I molecules, broadening the original oncoantigen spectrum targeted by Ab-based therapy. However, a limitation of TCRL mAb in this setting is low TCRL Ab coverage per cell due to MHC-I downregulation on tumors (30). In contrast, MHC-II is typically up-regulated on auto-APCs in autoimmunity. Further, the tight linkage of particular autoimmune diseases with particular MHC-II alleles (3) provides defined allelic targets for TCRL mAbs. Although depletion of pathology-driving cells, as in cancer therapy, is a therapeutic option in autoimmunity, TCRL mAb therapy typically aims to reestablish healthy immune balance among cells like CD4+ T_eff_ and T_reg_ cells by non-depleting mechanisms (**Figure 1**). Here, we propose a few options for future TCRL autoimmune therapeutics, based on advances in TCRL mAb cancer therapies (30, 31) and Ab therapies for autoimmune diseases (19, 88).

Antibody treatment can induce target cell apoptosis (89) or (via Ab Fc region) lead to antibody-dependent cell-mediated cytotoxicity (ADCC), antibody-dependent cellular phagocytosis (ADCP), or complement-dependent cytotoxicity (CDC) (88, 90) (**Figures 1A, B**). For example, anti-CD20 mAbs, FDA-approved for RA and primary progressive MS (91, 92), appear to work by depletion of CD20+ B cells, including those that present autoantigen to T cells and give rise to autoantibody-producing plasma cells. However, unclear long-term benefits and side effects (e.g., lack of vaccine Ab response) of broad B cell elimination are concerns (88). Alternatively, one may consider engineering a bispecific Ab (BsAb), coupling specificity of anti-CD20 and anti-autoantigen p/MHC for targeted depletion of pathology-related B cells (**Figure 1C**). In NOD mice, an autoimmune diabetes model, TCRL mAb alone were reported to delay diabetes onset, likely due to selective deletion of auto-APCs (54, 55); detailed mechanism and systemic immune impact await further investigation.

Non-depleting TCRL mAbs, for example those with low FcR binding (93, 94), provide additional avenues for therapeutic interventions. TCRL mAbs can limit autoantigen-MHC accessibility and reduce activation of cognate T cells (**Figure 1A**). This has long been the rationale for evaluating TCRL mAb specificity and functionality *in vitro* or in mouse models (37, 49, 50, 53, 54). Additionally, autoimmune modulators conjugated to or coupled with TCRL mAbs could facilitate modulator delivery to autoantigen-MHC-II-enriched sites of disease. Such modulators include toxins (**Figure 1D**), immunoregulatory cytokines, and antibodies that neutralize effector molecules or regulate effector cell activity (88). Cytokines like IL-10 and TGF-β that induce tolerogenic DC (95) with therapeutic efficacy (84) might reestablish tolerance at sites harboring auto-APCs (**Figure 1E**). Coupling TCRL mAb to FDA-approved antibodies that target inflammatory cytokines, as available for TNF, IL-6 and IL-1β, could localize their immunosuppressive effect to the sites of pathology (**Figure 1F**). TCRL mAbs could also be used in a chimeric antigen receptor (CAR) format for constructing CAR T cells (**Figure 1G**). In diabetic NOD mice, CAR T cells expressing an insulin peptide/MHC-II TCRL mAb modulated autoimmunity (54, 55). In addition, re-directing T_reg_ cells to the autoimmune milieu was shown to suppress autoreactive T_eff_ cells in several models (96). Thus, it may be fruitful to introduce TCRL CARs into T_reg_ cells for autoantigen-MHC directed T_reg_ cell activity (**Figure 1H**).

### Potential side effects of TCRL mAb therapy targeting autoantigen-MHC complexes

For TCRL mAbs that are on-target (specific for autoantigen-MHC) and on-tissue (targeting autoimmune lesion), their primary actions will be to deplete auto-APCs and/or to modulate the CD4+ T cell-mediated immune responses (**Figure 1**). However, adverse effects may arise after target auto-APC depletion or following immunomodulation. A potential concern with cell-depleting TCRL mAbs is autoantigen release from apoptotic auto-APCs, which may propagate autoimmunity (2). On-target but off-tissue or off-target binding by TCRL mAbs raises other risks, such as unpredictable cross-reactive interaction between these Abs and highly homologous HLA-II allelic proteins or mimetic self-peptides. For example, unexpected cross-reactivity of affinity-enhanced TCR reagents targeting cancer-related MAGE A3/HLA-A*01 complex was reported to result in fetal cardiotoxicity (29). Regardless of target specificity, immune activation or suppression subsequent to TCRL mAb administration may lead to unpredictable toxicities, such as new autoimmune reactions or reduced host defense. In general, most safety and side effect concerns associated with traditional Ab therapies (97, 98) are worthy of attention during TCRL Ab development and preclinical evaluation. To minimize the chance of causing adverse effects, efforts in 1) Fc engineering/modification to control Fc-mediated effector function, 2) advanced affinity maturation to avoid exaggerated/prolonged mAb binding to the target, and 3) rigorous immunopharmacology studies *in vitro* and in animal models (97), will be crucial at stages prior to clinical trials.

## Future directions

Despite great promise, effectively leveraging modern TCRL technologies in autoimmune therapy still requires optimization: First, advanced tools and innovative strategies for autoantigen discovery are still needed, as highly accurate identification and characterization of HLA-restricted peptide antigens are a prerequisite for downstream development of TCRL agents. Secondly, directed evolution and affinity maturation for low affinity TCRL candidates are still challenging, although combinatorial libraries designed using phage and yeast display platforms offer potential solutions. As more and more TCR and TCRL mAb structures emerge, machine learning (99) may offer more guidance on TCRL engineering. Last, for use in physiologic conditions, protein scaffolds other than mAbs sometimes possess better properties including protein stability, reduced immunogenicity, and increased tissue penetration (90, 100). Lessons learned from TCRL mAb development can be applied to alternative protein scaffolds (90) to expand TCRL methodology. Ongoing TCRL projects are focusing on resolving these issues, in hopes of opening an era for next generation autoimmune research and therapies.

## Author contributions

YL, WJ, and EM wrote the manuscript. All authors approved the submitted version.

## Conflict of interest

The authors declare that the research was conducted in the absence of any commercial or financial relationships that could be construed as a potential conflict of interest.

## Publisher’s note

All claims expressed in this article are solely those of the authors and do not necessarily represent those of their affiliated organizations, or those of the publisher, the editors and the reviewers. Any product that may be evaluated in this article, or claim that may be made by its manufacturer, is not guaranteed or endorsed by the publisher.
